# Investigating the Effect of Heat Treatment on the Microstructure and Hardness of Aluminum-Lithium Alloys

**DOI:** 10.3390/ma16196502

**Published:** 2023-09-30

**Authors:** Lida Radan, Victor Songmene, Yasser Zedan, Fawzy H. Samuel

**Affiliations:** Department of Mechanical Engineering, École de Technologie Supérieure, Montreal, QC H3C 1K3, Canada; lida.radan.1@ens.etsmtl.ca (L.R.); yasser.zedan.1@gmail.com (Y.Z.)

**Keywords:** aluminum-lithium (Al-Li) alloys, heat treatment, hardness, microstructure, precipitation strengthening

## Abstract

In this study, the effects of heat treatment on the microstructure and strength (micro-hardness) of an aluminum–lithium (Al-Li) base alloy containing copper (Cu) and scandium (Sc) were investigated, with a view to enhancing the alloy performance for aerospace applications. The heat treatment conditions were investigated to understand the precipitation behavior and the mechanisms involved in strengthening. Aging was carried out at temperatures of 130 °C and 150 °C for aging times of 1 h, 2.5 h, 5 h, 10 h, 15 h, 25 h, 35 h, and 45 h at each temperature for Al-Li alloy and at 160 °C, 180 °C, and 200 °C for aging times of 5 h, 10 h, 15 h, 20 h, 25 h, and 30 h at each temperature for Al-Li-Cu and Al-Li-Cu-Sc alloys. The investigation revealed that both solution heat treatment and artificial aging had a notable impact on strengthening the hardness of the alloy. This effect was attributed to the characteristics of the precipitates, including their type, size, number density, and distribution. The addition of copper (Cu) and scandium (Sc) was observed to have an impact on grain size refinement, while Cu addition specifically affected the precipitation behavior of the alloy. It led to remarkable changes in the number density, size, and distribution of T1 (Al_2_CuLi) and θ’ (Al_2_Cu) phases. As a result, the hardness of the alloy was significantly improved after the addition of Cu and Sc, in comparison with the base Al-Li alloy. The best heat treatment process was determined as: 580 °C/1 h solution treatment +150 °C/45 h artificial aging for Al-Li alloy and 505 °C/5 h solution treatment +180 °C/20 h artificial aging for Al-Li-Cu and Al-Li-Cu-Sc alloys.

## 1. Introduction

Aluminum is the most common material used in the aerospace industry because of its ease of availability, recyclability, and low density. Furthermore, adding lithium (Li) to aluminum (Al) as a lighter element creates great possibilities to enhance the overall performance of aircrafts [1,2,3].

The properties and performance of Al-Li alloys are influenced by a range of microstructural characteristics, including the size and shape of the grains, the formation of different phases at different places such as grain boundaries or within the grains, the nature of the precipitate-free zone (PFZ), and the overall texture of the material [4].

To enhance the strength of Al-Li alloys for structural purposes, several approaches are resorted to, including precipitation strengthening, solution strengthening, grain and sub-grain strengthening, and strengthening by dislocations [5].

The process of precipitation strengthening is recognized for its ability to strengthen these alloys and has generated significant interest in the development of new alloys for aerospace components, as indicated by recent research [6].

The microstructure of Al-Li alloys plays a crucial role in determining their macroscopic properties. As a result, the heat treatment process parameters can have a significant impact on the microstructure, allowing for potential modifications. These tailored microstructural features can ultimately enhance the mechanical properties of the alloy [7,8,9,10].

The temperature used for heat treatment plays an important role in the manufacturing of Al-Li alloys, as it can have a substantial impact on both the material’s microstructure and mechanical characteristics. The optimal heat treatment temperature can vary based on the specific Al-Li alloy and the desired properties. Generally, the heat treatment temperature for Al-Li alloys falls within the range of 130 °C to 600 °C, with solution and precipitation heat treatment being the most common methods employed. Solution heat treatment is a process employed for Al-Li alloys, where the material is heated to a temperature above the solidus temperature, typically between 500 °C and 580 °C [11,12]. Precipitation heat treatment, on the other hand, involves heating the Al-Li alloy to a temperature below the solidus temperature, usually between 120 °C and 200 °C. This process facilitates the formation of precipitates, which can boost the material’s strength and hardness [12].

Addition of elements and optimizing the precipitates are the main reasons for the excellent mechanical properties of Al-Li alloys [4]. Regarding the chemical composition of commercially available Al-Li alloys, copper (Cu) emerges as a significant alloying component in the Al-Li system. It imparts added strengthening impact by precipitating both T1 (Al_2_CuLi) and θ’ (Al_2_Cu) phases simultaneously, operating independently of the δ’ (Al_3_Li) phase as well. The preeminence of the T1 phase is recognized as the underlying reason for the exceptional mechanical attributes exhibited by these alloys [7].

Copper addition can also affect the size, distribution, and morphology of other precipitates in the Al-Li alloy [7,13]. For example, the δ’ (Al_3_Li) precipitates, which are also responsible for strengthening the alloy, can be refined, and dispersed more uniformly in the presence of copper [3].

Furthermore, alloying of Al-Li alloys with scandium (Sc) causes substantial grain refinement which increases strength characteristics, improves the weldability, improves high-temperature stability, and affects the nature and rate of decomposition of the supersaturated solid solution [14,15].

Experimental findings demonstrate that the precipitation sequence of an Al-Cu-Li alloy during solution treatment and artificial aging is influenced by the Cu/Li (wt%) ratio. Since in the current study, the composition of the Al-Li-Cu alloy reveals a Cu/Li ratio of less than 1, it is anticipated that the precipitation behavior of this alloy during solution and aging treatment will follow the sequence: SSS → δ’ + T1 → T1 [16].

In a study conducted by Deschamps et al. [17], the influence of the Cu/Li ratio on the precipitation sequence of Al-Cu-Li-X alloys was examined. According to their findings, during the early stages of aging, the microstructure of the Li-lean alloy predominantly consisted of clusters rich in copper (Cu), while the Li-rich alloy mainly exhibited the δ’ phase. Nevertheless, in both alloys, the microstructure observed at the peak-aged stage was predominantly characterized by the dominant presence of high-aspect-ratio T1 plates.

Nobel et al. [18] studied the structural aging characteristics of aluminum alloys containing 2 and 4 wt.% Li and they showed that the strengthening of these alloys is linked to the precipitation of the δ’ phase, where the greatest increase in hardness is observed in the 2% alloy after being aged at 200 °C for 24 h. At lower temperatures, the δ’ phase, which possesses a spherical shape, was extremely fine and exhibited resistance to coarsening.

In the research work of Lin et al. [9], the microstructure, hardness properties of an Al-2.58Cu-1.64Li alloy were examined under T6, T8 single aging, and T8 double aging conditions. The findings that were achieved after 48 h of secondary aging at 150 °C demonstrated that the highest hardness was obtained in the T8 duplex aging state, with a peak-aged hardness of 168 HV.

Xu et al. [19] conducted an investigation into the impact of aging treatments on the mechanical characteristics of Al-Cu-Li alloys. They aimed to elevate the quantity of the T1 phase through a dual approach involving pre-stretching and double aging. Samples subjected to these processes exhibited a faster rate of age hardening, reaching peak hardness in approximately 24 h at a temperature of 155 °C. The experimental results demonstrated a clear correlation between the pre-deformation, aging (PDA) temperature, and the rapid precipitation of the strengthening precipitate. The PDA processed sample exhibited the highest peak hardness, measuring 197.8 HV on the Vickers hardness scale [19].

Wu et al. [10] explored the changes in microstructure and concurrent mechanical properties of an Al-2Cu-2Li alloy as it underwent aging within the temperature of 150 to 225 °C. The findings suggested that the growth of the T1 phase was subject to the rate of Cu diffusion along dislocations.

Additionally, the initial hardness of the as-quenched alloy was approximately 125 HV. After aging at 175 °C for 32 h, the highest peak hardness of around 190 HV was achieved. Conversely, the alloy aged at 225 °C exhibited the lowest peak hardness, measuring approximately 142 HV [10].

In the research conducted by Suresh et al. [20], it was demonstrated that the addition of Sc and Zr elements to thermo-mechanically processed AA2195 Al-Li alloy resulted in the formation of Al_3_(Sc,Zr) particles. These particles played a crucial role in grain refinement and had a notable impact on the stability of the sub-grain structure.

Based on the literature review conducted above, it becomes evident that heat treatment has the potential to significantly enhance the mechanical properties of Al-Li based alloys. To date, despite the considerable research efforts dedicated to the heat treatment of Al-Li alloys, since the Al-Li-Cu and Al-Li-Cu-Sc alloys used in our study (synthesized from Al-4wt%Li master alloy) contain a high percentage of Li in addition to Sc and Cu, there is a definite need to find the appropriate heat treatments and investigate the precipitation strengthening behavior of these Al-Li based alloys. The present investigation aims to perform a series of heat treatment processes on these Al-Li based alloys and study the influence of heat treatment process parameters on the Vicker’s hardness and its microstructure.

## 2. Materials and Methods

**Figure 1:**
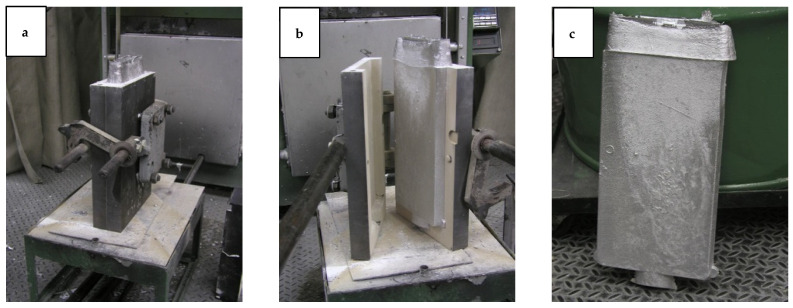
Casting of ingots of alloys used in the present study showing: (**a**) metallic mold with casting; (**b**) open mold showing casting inside; (**c**) actual casting.

The Al-Li alloy was received in the form of Al-4wt.%Li master alloy. Alloying elements were added in the form of pure Cu, Al-2wt.%Sc master alloy, and commercially pure Al (99.5%) to produce the compositions of the Al-Li, Al-Li-Cu, and Al-Li-Cu-Sc alloys shown in Table 1. The process for preparing the ingot castings for the three alloys studied is shown in Figure 1.

Different heat treatment schedules were designed to study the effect of the corresponding treatments on the microstructural features and micro-hardness of the three alloys. Samples (10 mm × 15 mm × 20 mm) sectioned from the casting ingot for each alloy were divided into three sets: for Al-Li alloy, one set was kept in the as-cast condition, while the second set was solution heat-treated at 580 °C/1 h, then quenched in warm water, and kept at −10 °C until testing. The third set was solution heat treated as before, quenched in warm water, followed by artificial aging at 130 °C and 150 °C for 1 h, 2.5 h, 5 h, 10 h, 15 h, 25 h, 35 h, and 45 h at each temperature. For the Al-Li-Cu and Al-Li-Cu-Sc alloys, one set was kept in the as-cast condition, the second set was solution heat-treated at 505 °C/5 h, then quenched in warm water, and kept at −10 °C until testing. The third set was solution heat treated as before, quenched in warm water, followed by artificial aging at 160 °C, 180 °C, and 200 °C for 5 h, 10 h, 15 h, 20 h, 25 h, and 30 h at each temperature. A summary of the heat treatment procedures is provided in Table 2. The solution and aging heat treatments were carried out in a Thermolyne Electric Furnace (Thermo Fisher Scientific, Waltham, MA, USA)

In all, 58 samples were used. Samples were mounted in bakelite using a Struers CitoPress-5 (Struers Limited, Mississigauga, ON, Canada) (force of 300 bars; heating time of 3 min, cooling time of 2 min). After being ground with standard SiC abrasive papers, the samples were mechanically polished in a sequence of 3 μm, 1 μm, and 0.5 μm diamond and subsequently electropolished in a Colloidal Silica—0.06 micron—Blue solution using a Buehler VibroMet™ 2 Vibratory Polisher (Buehler, Lake Bluff, IL, USA) for a duration of 24 h.

Vickers hardness measurements were carried out using an automatic CLEMEX micro-hardness tester (Clemex, Brossard, QC, Canada), using an indentation load of 100 gf applied for 10 s. Sixteen indentations were made on each specimen to analyze the hardness distribution. Scanning electron microscopic (SEM) analysis was carried out employing a Hitachi SU-8230 FE-SEM (Hitachi High-Tech Corporation, Ibaraki, Japan) equipped with a Bruker Quantax Flat Quad EDS detector (Bruker AXS LLC, Madison, WI, USA). X-ray diffraction (XRD) analysis was used to investigate phase evolution in the heat-treated and reference samples. The XRD experiments were carried out using a PANalytical X-ray diffraction machine model X’Pert Pro (Malvern Panalytical Ltd., Malvern, United Kingdom), using CuKα anode material, K-alpha 1 (Å): 1.54060, with step size (°2θ): n0.0170 and scan step time (s): 99.7000. The data obtained were analyzed by means of the associated X’Pert High Score software. Differential scanning calorimetry (DSC) analyses were performed using a DSC2500 Discovery series machine (TA Instruments, New Castle, DE, USA), with a scanning rate of 10 °C/min under argon atmosphere. Samples of approximately 9 mg were mechanically cut into squares of 1.9 mm × 1.9 mm × 1.2 mm size from the as-cast ingots and then solution treated. Alloy conditions for DSC analyses were as follows: Al-Li (solution treated at 580 °C for 1 h); Al-Li-Cu and Al-Li-Cu-Sc alloys (solution treated at 505 °C for 5 h). The samples were put into the alumina pan of the DSC machine; DSC runs were initiated at 40 °C and completed at 400 °C, and the samples cooled down from 400 °C to room temperature to examine the kinetics of phase transitions.

## 3. Results and Discussion

### 3.1. Hardness

**Figure 2:**
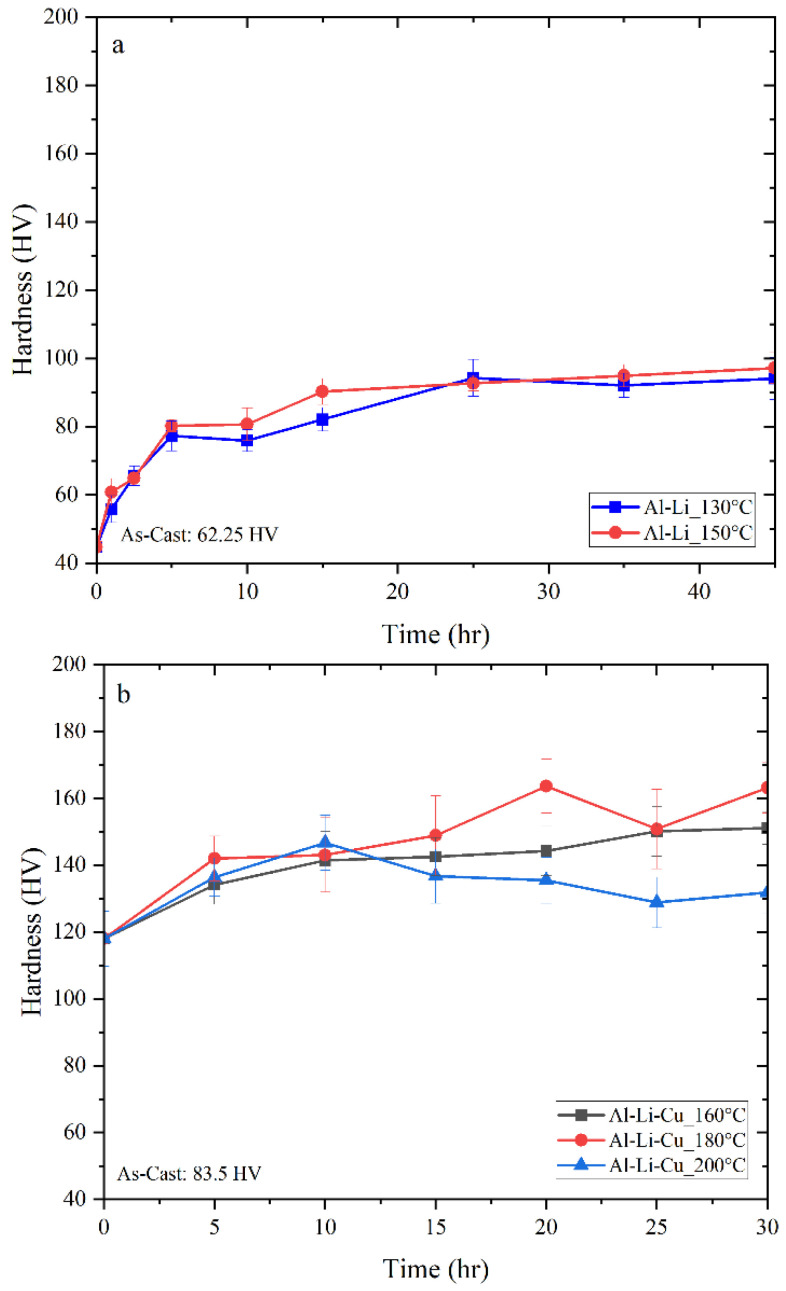

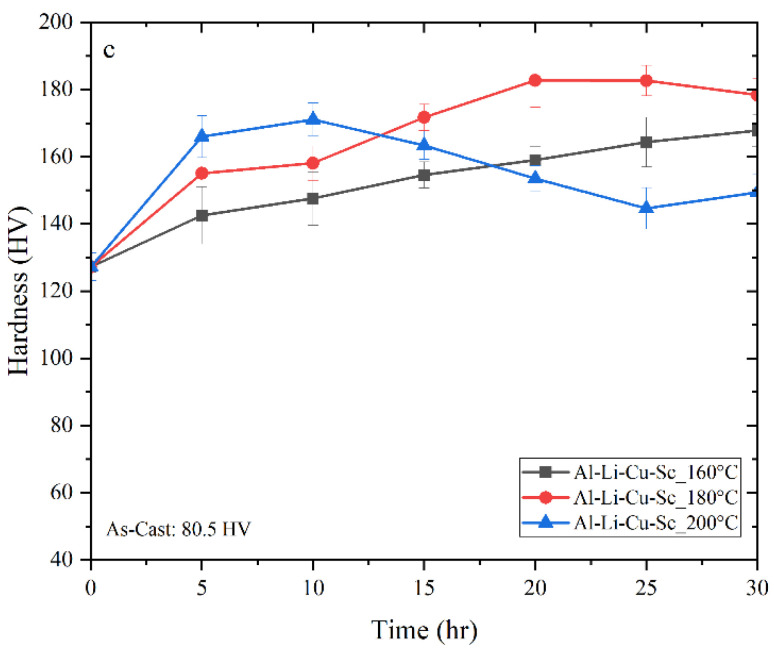
Variation in Vickers hardness values with aging time in samples of (**a**) Al-Li, (**b**) Al-Li-Cu, and (**c**) Al-Li-Cu-Sc alloys subjected to different heat treatment temperatures.

Figure 2 shows the development of hardness as a function of the natural aging time. The Al-Li alloy exhibits an increase in hardness with increase in aging time. Solution treatment for 1 h at 580 °C followed by artificial aging at 150 °C causes a change in hardness from 44.7 HV to its peak value of 97.2 HV after 45 h in Al-Li alloys. The primary cause of the increase in hardness observed in the Al-Li alloy was the formation of the δ’ phase.

The Al-Li-Cu and Al-Li-Cu-Sc alloys also show an increase in hardness with increase in aging time. For all heat treatment conditions, addition of Cu and Sc resulted in the highest peak-hardness. The strengthening effect of solution and aging treatments is the main reason for the change in hardness. Artificial aging at 180 °C for 20 h in Al-Li-Cu and Al-Li-Cu-Sc alloys led to peak-hardness values of 163.6 HV and 182.6 HV, respectively. Results of hardness values obtained for all three alloys are presented in Appendix A, Appendix B and Appendix C, respectively, at the end of the article.

Based on the binary Al-Li alloy diagram, the precipitation heat treatment process to enhance the strength and hardness of the material involves heating the Al-Li alloy to a temperature range of 130–200 °C, which facilitates the formation of precipitates. However, aging above 160 °C coarsens the precipitates; this has a negative effect on strength of the alloy. The δ’ phase that forms during the heat treatment process is the strengthening phase in Al-Li alloys and it plays a significant role in enhancing the alloy strength and stiffness. The actual degree of strengthening contributed by δ’ is a function of the volume fraction and the size distribution of the particles. When Al-Li alloys are heat-treated, the alloying elements (such as Li) diffuse through the aluminum matrix and combine to form the delta prime precipitates. While these precipitates are very small (typically less than 100 nm in size), they have a significant strengthening effect on the alloy. The precipitates act as obstacles to dislocation motion, making it more difficult for dislocations to move through the crystal lattice [21].

Both Al-Li-Cu and Al-Li-Cu-Sc alloys show a similar behavior and higher hardness because of the presence of Cu and hence the presence of copper aluminide phases such as T1 and θ’. In addition, the precipitation of a large number of phases such as T1, θ’, and δ’, directly contributes to the rapid rise in hardness. Utilizing elevated temperatures can speed up the diffusion path of solute atoms. However, this acceleration might induce untimely growth of the strengthening phases, ultimately resulting in a decline in the nucleation density of precipitates. Consequently, such an outcome would have a detrimental impact on the hardness, or strength of the alloy.

In the course of solution treatment, the secondary phases in the alloy dissolve into the matrix, leading to the creation of a supersaturated solid solution. During the aging process, the nature of precipitates within the alloy undergoes transformations and changes which influence the alloy’s characteristics. These variations in the precipitates ultimately contribute to the alterations observed in the hardness of the alloy [17,22].

In addition to the named precipitates, the Al-Li-Cu-Sc alloy containing Sc exhibited the highest hardness values. This was ascribed to the presence of Al_3_Sc precipitates, which, having a very small size (10–100 nanometers), creates a high density of obstacles for dislocations to move through. Furthermore, the addition of Sc to aluminum alloys promotes the formation of a high density of fine, uniformly distributed precipitates within the grains which further contribute to the strengthening [14,20].

### 3.2. SEM Characterization

SEM images of aluminum lithium alloys can provide a detailed view of the microstructure of the material, including the distribution and morphology of precipitates. The precipitates present in Al-Li alloys are typically small and can range in shape from spherical to elongated or needle-like.

**Figure 3:**
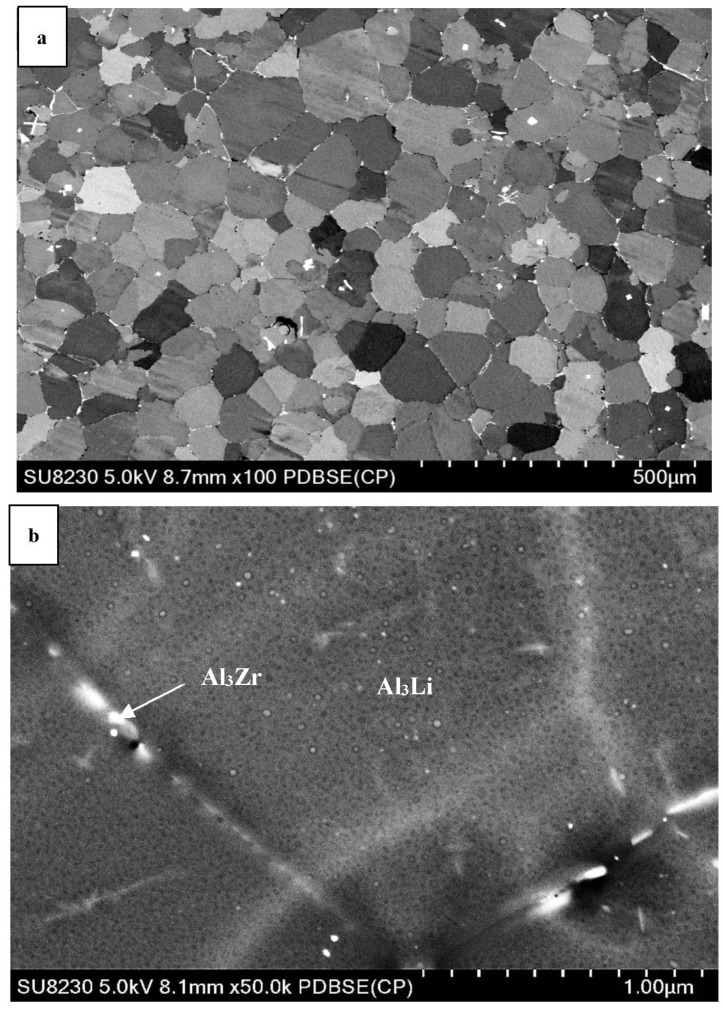

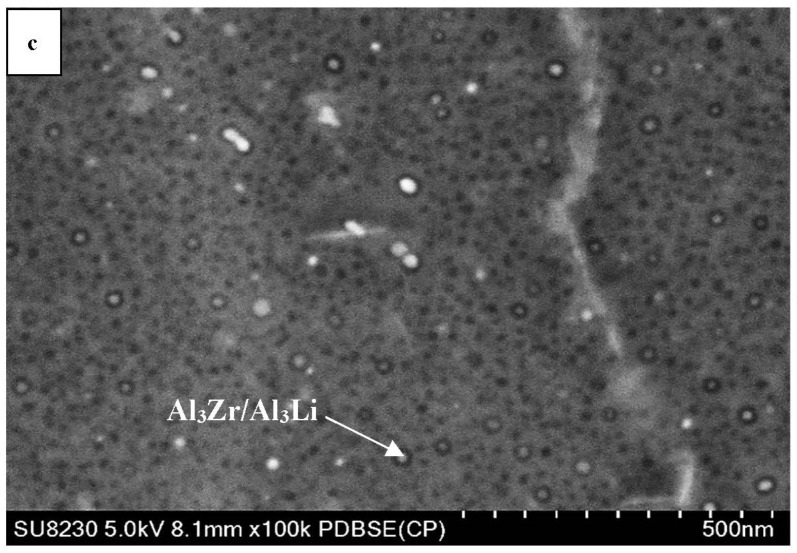
SEM micrographs of aged Al-Li alloy, showing (**a**) the grain structure; (**b**) precipitation of Al_3_Zr at the grain boundaries; (**c**) precipitation ofAl_3_Li within the matrix.

**Figure 4:**
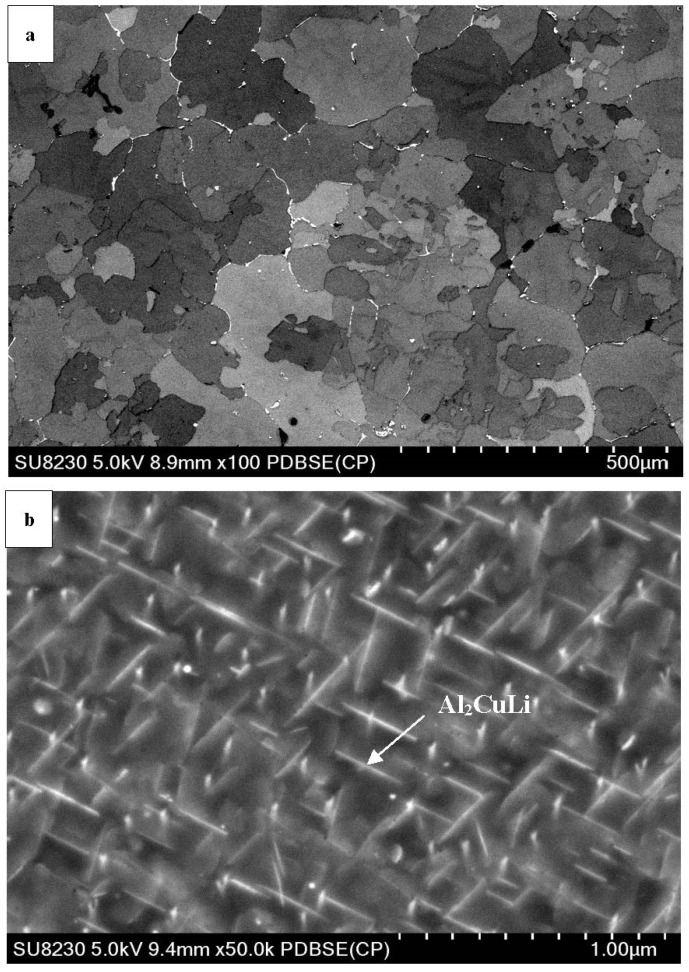
SEM micrographs of aged Al-Li-Cu alloy showing (**a**) general view of Al grains with precipitates at the grain boundaries; (**b**) precipitation of AlCuLi(arrowed).

**Figure 5:**
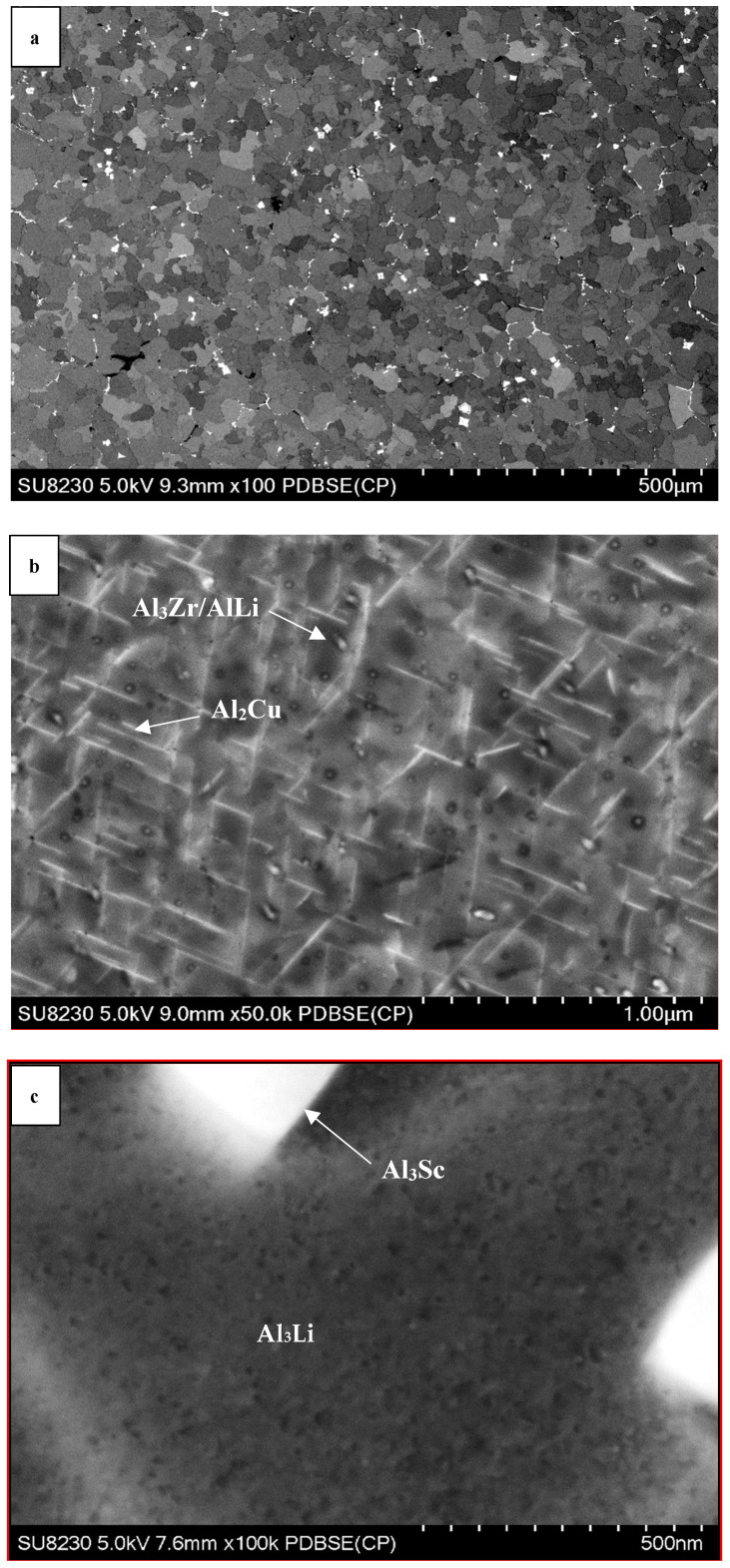
SEM micrographs aged Al-Li-Cu-Sc alloy showing (**a**) general grain structure; (**b**) high magnification image showing Al_3_Zr/AlLi and Al_2_Cu precipitated (arrowed; (**c**) precipitation of Al_3_Sc dispersoid in the matrix.

Figure 3, Figure 4 and Figure 5 display various SEM micrographs of the three alloys studied. Figure 3a depicts Al-Li alloy after aging (low magnification image) which shows δ (AlLi) precipitates that mainly appear at the grain boundaries of the α-Al with homogenous microstructure. In Figure 3b, it can be seen that the Al_3_Zr precipitates are mostly dispersed in grain boundaries and form as discontinuous networks or clusters. They act as effective barriers to dislocation movement, impeding plastic deformation and enhancing the alloy’s strength. Overall, the Al_3_Zr precipitates in grain boundaries of Al-Li alloys contribute to strengthening the material and promoting a fine-grained microstructure. These effects result in improved mechanical properties and increased performance in various applications [23]. The presence of spherical β’ facilitates the initiation of nucleation sites for δ’ leading to the formation of a distinctive pattern known as the bull’s-eye structure [24]. The ‘bull’s-eye’ type features in Figure 3c indicate the spherical form of the precipitates.

Comparing Figure 3a, Figure 4a and Figure 5a shows that adding Cu to the Al-Li alloy changes the size of the grain in the microstructure. Copper atoms tend to segregate to grain boundaries in aluminum alloys and act as a grain refiner in aluminum lithium alloys, but in Figure 4a, the grains grow unexpectedly. The presence of copper at the grain boundaries may hinder grain boundary mobility and promote grain growth. This can lead to larger grain sizes compared to the pure aluminum lithium alloy. The grain size is reduced by adding Sc to the alloy (see Figure 5a). In addition to that, the presence of Cu and Sc to the Al-Li alloy causes the precipitates to distribute within the matrix rather than being precipitated at the grain boundaries. During the aging treatment, the alloy forms several precipitates, namely T1 (Al_2_CuLi), θ’ (Al_2_Cu), β’ (Al_3_Zr), and δ’ (Al_3_Li) phases. Among these, the T1 phase is the one that impedes dislocation movement effectively, attributed to its non-shearing properties.

The higher magnification images presented in Figure 4b and Figure 5b show that the shapes of the precipitates are changed after adding Cu and Sc to the Al-Li alloy. Tiny spherical precipitates are visible in the microstructure of the Al-Li-Cu-Sc alloy as may be seen in Figure 5b. It seems that the larger plate-like phases precipitate at the grain boundaries, whereas the smaller ones appear within the grains. Incorporating Sc into the alloy increases the density of precipitates and encourages a more uniform dispersion of these particles. Additionally, it is widely acknowledged that Sc significantly improves the mechanical properties of aluminum alloys by facilitating the formation of the Al_3_Sc phase through precipitation (Figure 5c). The dispersion of particles of Al_3_Sc enhances the strength, substantially reinforces the substructure, and hinders the process of recrystallization [20].

Scandium (Sc) exhibits low solubility in aluminum and serves as a potent structure modifier in Al-Li alloys. It also displays an anti-recrystallization effect [15].

**Figure 6:**
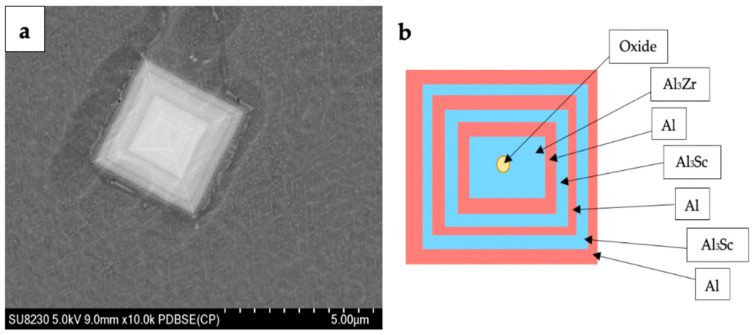
(**a**) SEM image of the morphology of an Al_3_(Sc,Zr) particle; (**b**) Diagrammatic sketch of the structure of the layered particle.

According to the studies conducted by Norman et al. [25] and Hyde et al. [26], it has been suggested that primary Al_3_(Sc,Zr) particles possess a quasi-cubic morphology, while their two-dimensional shape appears to be rectangular. The SEM micrograph in Figure 6a reveals a triangular-shaped Al_3_(Sc,Zr) particle. The distribution of alloying elements is heterogeneous. The particle exhibits a layered structure, where each layer has a distinct composition. Some layers are enriched in Sc or Zr but depleted in Al, while other layers are enriched in Al but depleted in Sc and Zr. A diagrammatic representation of the layered structure of the rectangular particle is provided in Figure 6b.

The formation of the layered structure in these particles follows a specific sequence of reactions. Initially, central oxides act as nucleation sites for the Al_3_Zr phase, making it the first phase to form. Subsequently, the first layer of aluminum (Al) is formed through the reaction Al_3_Zr + liquid → Al. Following this, Al_3_Sc is formed on the Al layer via the reaction liquid → α-Al + Al_3_Sc. The close orientation relationship and small lattice mismatch between Al and Al_3_Sc facilitate the easy nucleation of Al_3_Sc on Al. As a result, alternating eutectic reactions occur between Al_3_Sc on Al and Al on Al_3_Sc, leading to the formation of a multi-layered Al_3_(Sc,Zr) phase particle. Consequently, the multi-layer structure consisting of Al_3_Zr + α-Al + Al_3_Sc + α-Al + Al_3_Sc + … is developed [26,27,28,29].

### 3.3. X-ray Diffraction (XRD) Analysis

**Figure 7:**
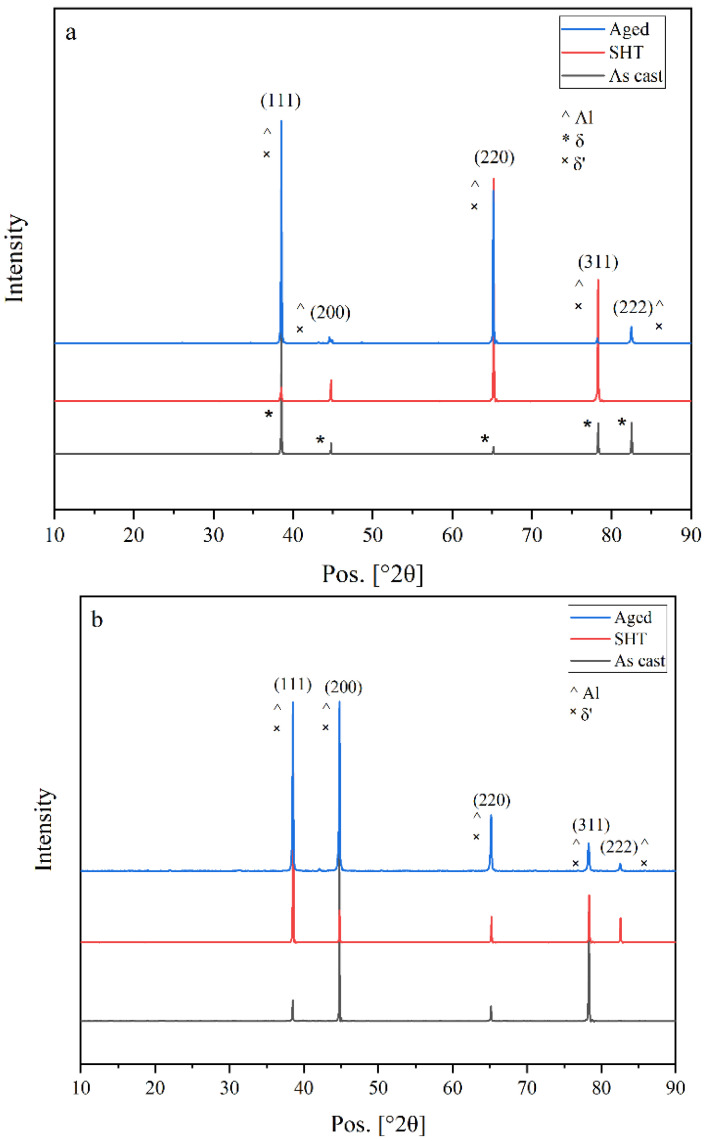

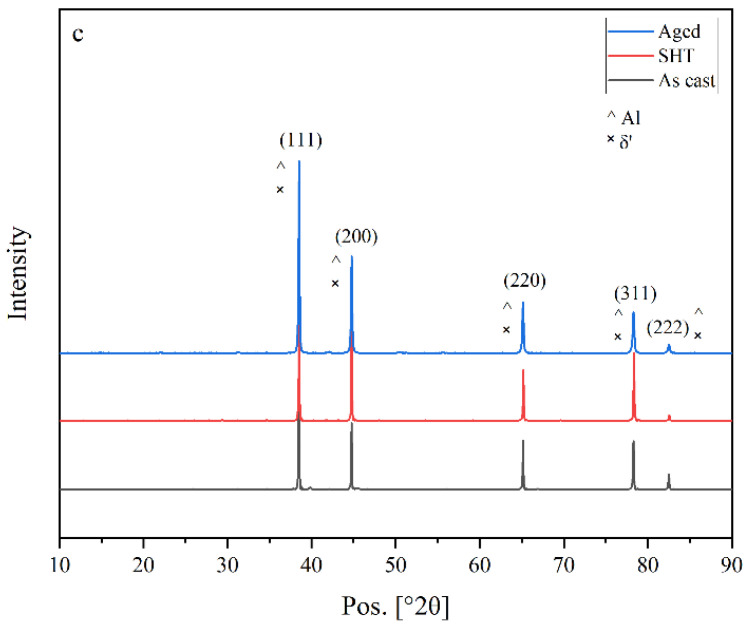
XRD patterns of: (**a**) Al-Li alloy in as-cast, solution heat treated (580 °C/1 h) and aged (SHT + 150 °C/45 h) conditions; (**b**) Al-Li-Cu alloy in as-cast, solution heat treated (505 °C/5 h) and aged (SHT + 180 °C/20 h) conditions; (**c**) Al-Li-Cu-Sc alloy in as-cast, solution heat treated (505 °C/5 h) and aged (SHT + 180 °C/20 h) conditions.

Since the atomic number of lithium is low, EDS cannot be used to detect the distribution of Li and its constituent phases. Therefore, to evaluate the influence of heat treatment on the phase types in the alloys investigated, the X-ray diffraction (XRD) technique was used. Figure 7 presents a synopsis of the diffraction peaks observed for the as-cast, solution heat-treated and aged samples of Al-Li, Al-Li-Cu, and Al-Li-Cu-Sc alloys as an example.

In the Al-Li alloys, based on the XRD phase analysis and detection results, it was observed (Figure 7a) that the predominant Li-containing alloy phases present in the Al-Li alloy were α-Al, AlLi, and Al_3_Li phases. Furthermore, it was found that these phases remained unchanged even after subjecting the alloy to heat treatment. In the as-cast alloy, α-Al and AlLi phases were the main peaks. Most intermetallic phases that dissolved into the Al matrix cause the decline of the diffraction peaks of the secondary phases after solution heat treatment. The δ (AlLi) phase disappeared after aging (SHT + 150 °C /45 h) and the δ’ (Al_3_Li) phase was precipitated and appeared.

As may be seen from Figure 7b, while different secondary phases were observed in the as-cast alloy, α-Al and Al_3_Li phases were the main peaks noted. In the context of previous studies [30], other phases such as Al_2_Cu, AlLi, and Al_6_CuLi_3_ should also exist in the as-cast alloy, but no visible peaks of these phases were discerned in the present sample, possibly because of their relatively small amounts. According to Noble and Bray [31], the intermetallic phase δ’-Al_3_Li is present in all the as-cast alloys due to the decomposition of the α-Al supersaturated solid solution during the solidification process.

The obvious decline of the diffraction peaks of the secondary phases after solution heat treatment is the most significant aspect, which indicates that most intermetallic phases have dissolved into the Al matrix. Following the solution treatment, the AlLi phase disappeared, and a few Al_2_Cu phase particles were generated. Subsequently, during the aging process (SHT + 200 °C /20 h), precipitation of the T1, θ’, and δ’ (Al_3_Li) phases was observed.

It was observed that the predominant phases in Al-Li-Cu-Sc alloy were α-Al, Al_3_Li, θ’, and T1 phases. In the as-cast alloy, α-Al, δ (AlLi), and T1 phases were the main peaks. The δ (AlLi) intermetallic phase dissolved into the Al matrix after solution heat treatment. The δ (AlLi) phase disappeared after aging (SHT + 180 °C /20 h), and the T1, θ’, and δ’ (Al_3_Li) phases were seen to precipitate. In Al-Li-Cu-Sc alloy, other phases which contain Sc are expected to exist in all conditions, but no visible peaks of these phases were detected in the present sample, possibly because of their relatively small amounts in the alloy.

### 3.4. DSC Investigation

**Figure 8:**
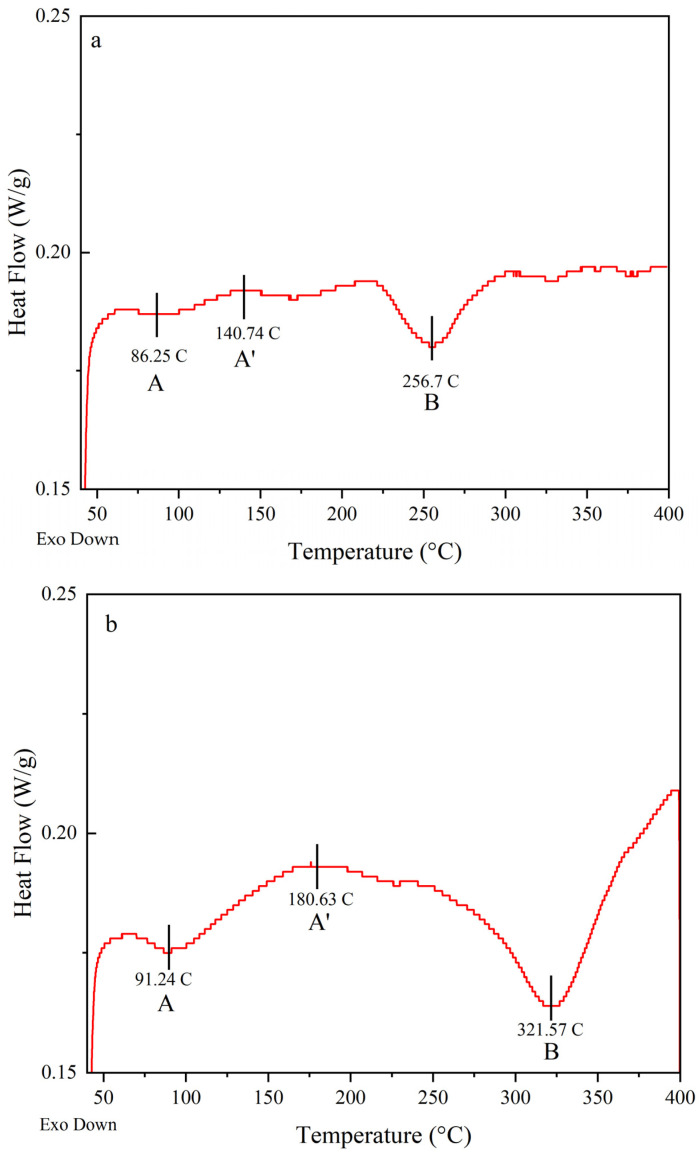

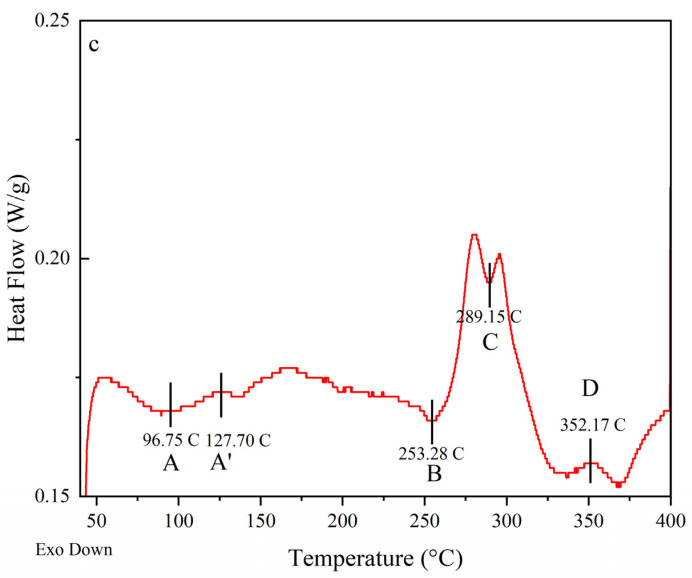
The DSC analysis was performed on the as-quenched (solutionized) samples of all compositions and obtained at 10 °C/min. (**a**) DSC heating curve of the solutionized Al-Li alloy; (**b**) DSC heating curve of the solutionized Al-Li-Cu alloy; (**c**) DSC heating curve of the solutionized Al-Li-Cu-Sc alloy. A, B, C represent the onset temperatures of precipitation (Tonset), while A’ and D represent the peak temperatures of precipitation (Tpeak) of corresponding reactions. The red curves show the DSC heating curve in each case.

The thermal behavior and phase transformations in an alloy are influenced by various factors, including the alloy composition, heat treatment, and the presence of impurities. Figure 8 illustrates the DSC curves acquired for the as-quenched samples of the studied alloys, Al-Li, Al-Li-Cu, and Al-Li-Cu-Sc, at a heating rate of 10 °C min^−1^.

For Al-Li alloys, a commonly observed exothermic peak occurs during the precipitation of the strengthening phase, which is usually Li-rich. The peak associated with this phase precipitation can typically be found in the temperature range of 100–250 °C, depending on the specific alloy composition. After solution treatment, upon cooling or during the early stages of aging, the formation of Guinier–Preston (GP) zones occurs. As aging progresses, the GP zones grow and transform into more coherent and strengthening precipitates of δ’ (Al_3_Li). The formation of δ’ precipitates occurs through a nucleation and growth process, leading to the clustering of Li atoms. The temperature range for δ’ precipitate formation typically occurs in the range of 180–250 °C [32,33,34].

In Figure 8a, the exothermic peak A appears to correspond to the formation of GP zones, while the small endothermic peak A’ around ~150 °C likely indicates the dissolution of GP zones. The presence of peak B at approximately 250 °C can be attributed to the formation of the δ’ phase.

Aging in Al-Li-Cu alloys typically involves the formation of GP zones, which are clusters of Cu atoms within the aluminum matrix. The copper aluminide precipitates are usually Al_2_CuLi intermetallic compound. The exact temperature range in which this precipitate forms is typically within 120–250 °C. Further aging at higher temperatures leads to the transformation of copper aluminide precipitates into a more stable phase θ’ (Al_2_Cu). The Al_2_CuLi (T1) precipitates are usually Al_2_CuLi intermetallic compounds with a refined and ordered structure. The temperature range for this precipitate formation typically occurs in the range of 160–250 °C. In some Al-Li-Cu alloys, the aging process can also lead to the formation of δ’ precipitates [32,35].

In Figure 8b, the DSC curve for the Al-Li-Cu alloy exhibits an exothermic peak at 100 °C (Peak A) that represents the formation of GP zones. The dissolution of GP zones is observed in the second peak, peak A’. The exothermic reaction corresponding to peak B is attributed to the precipitation and growth of θ’, T1, and δ’ phase precipitates. After solution treatment, during the early stages of aging, the formation of GP zones occurs. As aging progresses, the GP zones undergo a transformation and grow into coherent and strengthening θ’ (Al_2_Cu) precipitates. The presence of Sc influences the precipitation process and can affect the size, distribution, and stability of the θ’ precipitates.

In Al-Li-Cu-Sc alloys, the addition of Sc promotes the formation of Al_3_Sc precipitates. The formation of the Al_3_Sc phase in Al-Li-Cu-Sc alloys generally occurs at temperatures ranging from approximately 300 °C to 400 °C [33,36]. Figure 8c is the DSC curve for a quenched Al-Li-Cu-Sc alloy specimen. The formation of GP zones is indicated by the exothermic peak A. Additionally, a small endothermic peak A’ occurring around ~125 °C is observed, likely associated with the dissolution of the GP zones. The formation of the δ’ phase can be attributed to peak B at approximately 260 °C. The exothermic reaction corresponding to peak C is ascribed to the precipitation and growth of θ’ and T1 phases. The last peak D, at ~370 °C, is expected to correspond to the formation of Al_3_Sc precipitates.

## 4. Conclusions

This study focused on the heat treatment of Al-Li, Al-Li-Cu, and Al-Li-Cu-Sc alloys to investigate their microstructure and microhardness under different heat treatment processes. A detailed examination of the alloy samples was carried out to analyze the influence of these processes. Based on the findings from SEM, XRD, and DSC analyses, as well as micro-hardness measurements, the following conclusions were drawn.

1. The main strengthening phase in Al-Li alloy is the δ’ (Al_3_Li) phase, while in Al-Li-Cu and Al-Li-Cu-Sc alloys, the main phases that increase hardness are the δ’ (Al_3_Li) phase, the T1 (Al_2_CuLi) phase, and the θ’ (Al_2_Cu) phase. The dispersion of small spherical particles of Al_3_Sc in Al-Li-Cu-Sc alloy enhances the hardness, substantially reinforces the substructure, and hinders the process of recrystallization.
2. Adding Cu and Sc contributes to a minor reduction in grain size while also generating supplementary secondary phases enriched with Cu. Throughout the aging process, precipitation and the subsequent transformation of precipitates could enhance the alloy’s strength more. Particularly, the density of Cu-enriched precipitates, predominantly T1, exhibits an increase by adding Cu content during aging. The outcomes derived from this study have the potential to offer empirical data and a point of reference for subsequent inquiries, which might contribute to advancing the development and potential applications of Al-Li alloys containing 2–3 wt.% Li, alongside the inclusion of Cu and Sc.
3. The hardness of the Al-Li-Cu and Al-Li-Cu-Sc alloys increases rapidly with aging time and reaches a maximum, and then decreases slowly. The hardness of these two alloys exhibits an initial increase followed by a slight decrease during the aging process.
4. The optimal heat treatment process is determined to be as follows:
  - 580 °C/1 h solution heat treatment +150 °C/45 h artificial aging for Al-Li alloy;
  - 505 °C/5 h solution treatment +180 °C/20 h artificial aging for Al-Li-Cu and Al-Li-Cu-Sc alloys.

Under these conditions, the maximum hardness of the heat-treated Al-Li, Al-Li-Cu and Al-Li-Cu-Sc alloys are, respectively, 97.2 HV, 163.6 HV, and 182.6 HV.

Hence, the findings acquired through this study have the potential to provide empirical data and serve as a reference for extended explorations, and potentially develop the advancement and utilization of Al-Li alloys comprising 2–3 wt.% Li, in addition to the incorporation of Cu and Sc.

## Figures and Tables

**Table 1:** Chemical compositions of the Al-Li alloys used in this study.

Element (wt%) *													
Alloy	Si	Mg	Cr	Mn	Fe	Cu	Zn	Ni	Ti	V	Li *	Zr	Sc
Al-Li	0.58	<0.003	<0.003	0.027	0.28	0.23	0.040	0.010	0.052	0.012	1.9	0.3	<0.010
Al-Li-Cu	0.18	<0.003	<0.003	0.012	0.099	2.9	0.039	0.007	0.090	0.010	3.0	0.3	<0.010
Al-Li-Cu-Sc	0.17	<0.003	<0.003	0.011	0.10	2.6	0.039	0.007	0.067	0.010	2.9	0.3	0.185

* Chemical composition was determined using optical emission spectroscopy.

**Table 2:** Heat treatments applied to the used Al-Li alloys.

Alloy	Solution Heat Treatment Temp (°C)/Time (h)	Aging Temp. (°C)	Aging Time (h)
Al-Li	580 °C/5 h	130, 150	1, 2.5, 5, 10, 15, 25, 35, 45
Al-Li-Cu	505 °C/5 h	160, 180, 200	5, 10, 15, 20, 25, 30
Al-Li-Cu-Sc	505 °C/5 h	160, 180, 200	5, 10, 15, 20, 25, 30

## Data Availability

Data will be made available upon request.

## Appendix A

**Table A1:** Al-Li alloy.

No.	Solutionizing Temperature (°C)	Solutionizing Duration (h)	Aging Temperature (°C)	Aging Duration (h)	Vickers Hardness (VHN)
1	580	1	130	1	55.8
2	580	1	130	2.5	65.6
3	580	1	130	5	77.3
4	580	1	130	10	75.9
5	580	1	130	15	82.1
6	580	1	130	25	94.3
7	580	1	130	35	92.1
8	580	1	130	45	94.1
9	580	1	150	1	60.8
10	580	1	150	2.5	64.9
11	580	1	150	5	80.2
12	580	1	150	10	80.7
13	580	1	150	15	90.3
14	580	1	150	25	92.7
15	580	1	150	35	94.9
16	580	1	150	45	97.2

## Appendix B

**Table A2:** Al-Li-Cu alloy.

No.	Solutionizing Temperature (°C)	Solutionizing Duration (h)	Aging Temperature (°C)	Aging Duration (h)	Vickers Hardness (VHN)
1	505	5	160	5	134.125
2	505	5	160	10	141.4375
3	505	5	160	15	142.5625
4	505	5	160	20	144.25
5	505	5	160	25	150.125
6	505	5	160	30	151.125
7	505	5	180	5	142.01
8	505	5	180	10	143.0625
9	505	5	180	15	148.875
10	505	5	180	20	163.625
11	505	5	180	25	150.8125
12	505	5	180	30	163.1875
13	505	5	200	5	136.375
14	505	5	200	10	146.6875
15	505	5	200	15	136.6875
16	505	5	200	20	135.4375
17	505	5	200	25	128.8125
18	505	5	200	30	131.875

## Appendix C

**Table A3:** Al-Li-Cu-Sc alloy.

No.	Solutionizing Temperature (°C)	Solutionizing Duration (h)	Aging Temperature (°C)	Aging Duration (h)	Vickers Hardness (VHN)
1	505	5	160	5	142.5
2	505	5	160	10	147.5625
3	505	5	160	15	154.5625
4	505	5	160	20	159
5	505	5	160	25	164.3125
6	505	5	160	30	167.75
7	505	5	180	5	155.034
8	505	5	180	10	158.0625
9	505	5	180	15	171.6875
10	505	5	180	20	182.6875
11	505	5	180	25	182.625
12	505	5	180	30	178.375
13	505	5	200	5	165.9375
14	505	5	200	10	171.0625
15	505	5	200	15	163.375
16	505	5	200	20	153.4375
17	505	5	200	25	144.625
18	505	5	200	30	149.375
